# WORKING WITH DIVERSE ELDERS IN RURAL COMMUNITIES (OPPORTUNITIES AND STRENGTHS)

**DOI:** 10.1093/geroni/igae098.1706

**Published:** 2024-12-31

**Authors:** Karen Kopera-Frye, Lyn Holley, Cassandra Ford

**Affiliations:** Chair; Co-Chair; Discussant

## Abstract

The focus of this symposium is to discuss the opportunities and strengths of working with diverse elders and older adults in rural communities. Accounts of field experiences will be shared, and methods of utilizing existing data that hold promise for addressing current problems; along with building the foundation of communication and relationship requisite to successful design, development, and implementation of improvements will be described through various studies with rural and/or indigenous peoples. We will discuss the role of social determinants of health, race, and rurality on frailty trajectories utilizing data from the National Health and Aging Trend Study. An innovative approach for leveraging older adult farmers’ expertise to facilitate effective emergency decision-making in Fraiser Valley, British Columbia, Canada, will be described. Ongoing research with Alaskan Native Elders provides insight into the need for effective research with Indigenous cultures, especially Elders, by developing trusting and mutually beneficial relationships. Research strategies involved historical, social, cultural, and inter-relational perspectives. Insight into how Cuidando con Respeto, a program with Latinx elders, builds bridges to health care and mutual understanding nurtured at the community level and shows promise in empowering caregivers within a familismo-culturally relevant manner will be shared. The use of mobile pain applications and considerations when designing health technology will be examined with diverse older adults.

